# Walking Recovery After Hip Fracture in Patients with Exceptional Longevity: Predictors and Association with Short- and Long-Term Survival

**DOI:** 10.3390/jcm15135085

**Published:** 2026-06-30

**Authors:** Montserrat Barceló, Patricia Valentina Marquez, Ignasi Gich, Jordi Casademont, Olga H. Torres

**Affiliations:** 1 Departament de Medicina, Universitat Autònoma de Barcelona, 08193 Barcelona, Spain; pmarquez@santpau.cat (P.V.M.); igichs@santpau.cat (I.G.); jcasademont@santpau.cat (J.C.); 2 Institut de Recerca Sant Pau (IR Sant Pau), 08041 Barcelona, Spain; olga.torres@uab.cat; 3 Geriatrics Unit, Internal Medicine Department, Hospital de la Santa Creu I Sant Pau, Mas Casanovas Street n° 90, 08041 Barcelona, Spain; 4 CIBER Epidemiología y Salud Pública (CIBERESP), 28019 Madrid, Spain

**Keywords:** mortality, hip fracture, centenarians, exceptional longevity, oldest-old

## Abstract

**Background:** There are very few studies on walking recovery, its predictors and impact on survival in oldest-old patients after a hip fracture. **Methods:** This study is a retrospective review which included all patients older than 95 years admitted with a fragility hip fracture between December 2009 and September 2015 in a tertiary university hospital in Barcelona. Walking ability was assessed using the Functional Ambulation Classification (FAC) prior to admission and 6 months after discharge. The objective of our study is to assess walking recovery and its predictors in oldest-old patients at 6 months after discharge, and to determine whether there was a relationship with short and long-term survival. **Results:** One hundred and fifty-two patients were included in the study. Prior to the fracture, 78.3% of patients could walk independently, 36.8% after the fracture. A higher previous FAC score (*p* < 0.001, OR 3.658), absence of delirium during admission (*p* = 0.010, OR 3.45), and being able to carry out full weight-bearing (*p* = 0.026, OR 12.705) were associated with better walking recovery. The area under the ROC curve was 0.819 (*p* < 0.001). Mean survival after discharge was 2.24 years (SD 1.185). Patients with a post-fracture FAC ≥ 3 showed better survival. **Conclusions:** Predictors of walking recovery in patients with exceptional longevity were a higher previous FAC score, being able to carry out full weight-bearing, and absence of delirium. Patients able to walk unaided within six months of discharge showed better survival. These findings highlight the importance of functional outcomes when assessing prognosis in the oldest-old.

## 1. Introduction

Although people with extreme longevity represent a small proportion of the population, their number is increasing rapidly in Western countries. A 2017 study described this increase in the European Union, where the number of centenarians doubled in 5 years [1]. Centenarians are at high risk of fragility fractures. In Spain, the incidence of hip fractures in centenarians is about 4%, which is seven times higher than in younger age groups [2]. Hip fractures in older adults are significant because they can severely impair or lead to a loss of functional abilities [3,4].

Several studies have evaluated outcomes after following hip fractures. Although the results vary considerably, it is generally reported that a significant percentage of patients do not regain the ability to walk. A study by Gonzalez Marcos [5] found that 45% of individuals over the age of 65 were unable to recover walking ability. Predictors of walking recovery in patients with hip fractures were identified as younger age, independent walking ability prior to the fracture, and absence of cognitive impairment [6,7,8].

Very few studies have examined walking recovery in extremely old patients following a hip fracture. Most of these studies included small numbers of patients and the percentage of patients who regained mobility was low, in some cases less than 25% [9,10]. However, other studies have reported better walking recovery rates of around 50% or more [11,12,13], although functional loss is greater than in younger age groups: 74% of octogenarians and 65% of nonagenarians regained unassisted walking [11]. Furthermore, in younger patients with a mean age of less than 80 years, those able to ambulate after discharge had better one-year and 10-year survival [14,15]. No studies have explored the relationship between walking ability and survival in the exceptional longevity age group. Assessment of ambulatory recovery prospects is particularly relevant in the oldest-old population. Accurate prognostic information can help ensure that rehabilitation and physiotherapy efforts are maintained, especially in patients with a higher likelihood of functional recovery. It also facilitates care planning and supports patients and their families in making informed decisions regarding future support needs and living arrangements.

We hypothesize that walking recovery in patients with exceptional longevity may be as high as 50%, as has been reported previously, and that the predictors of recovery will be similar to those observed in younger individuals. Furthermore, we predict that patients who demonstrate better walking recovery will also show better survival.

## 2. Methods

### 2.1. Setting

The study was conducted in the orthopedic surgery ward of the Hospital de la Santa Creu i Sant Pau, a tertiary university hospital in Barcelona serving a population of approximately 425,000 people with a high proportion of older adults.

### 2.2. Subjects

All patients older than 95 years discharged from the orthopedic surgery ward with a diagnosis of fragility hip fracture between December 2009 and September 2015 were retrospectively reviewed. Survival was assessed up to the present time or the patient’s date of death. Patients with hip fractures resulting from a traffic accident or neoplasms were excluded. Patients for whom walking ability was not documented, and those who died within one month of discharge and before the first appointment with the traumatologist, were also excluded because their walking ability had not been recorded.

This study is a continuation of a previous study in patients with exceptional longevity [16] and was approved by the institutional ethics committee (code IIBSP-FEM-2023-36). The study was conducted in accordance with the ethical principles of the Declaration of Helsinki for medical research involving human subjects.

During hospitalization, a multidisciplinary approach was followed with the participation of a physician from the Geriatric Unit. Bed rest was imposed for 24 h after surgery, and physiotherapy was started 24–48 h after surgery, if the patient was clinically stable. A delirium assessment was performed during all three nursing shifts.

### 2.3. Data Collection

The following data were collected retrospectively: age, sex, comorbidities, as measured by the Charlson Comorbidity Index [17], dementia, depression diagnosed before admission, pre-fracture and post-discharge ambulation, as measured by the Functional Ambulation Classification (FAC) [18], place of residence, number of medications prior to admission, and hemoglobin and albumin levels at admission. The following were also recorded: length of hospital stay, delay to surgery, type of fracture (intracapsular or extracapsular), type of surgery (with or without prosthesis), type of anesthesia, medical and traumatological complications during admission (including delirium and transfusion), full weight-bearing capacity after surgery and final discharge destination.

Regarding the type of fracture, subcapital and basicervical hip fractures were considered intracapsular, while pertrochanteric fractures were classified as extracapsular. Regarding the type of surgery, hemiarthroplasty was considered a prosthetic procedure, whereas cannulated screws, sliding hip screws, osteosynthesis and intra-medullary nails were classified as non-prosthetic procedures.

The post-discharge FAC was used to assess the maximum level of walking recovery achieved by the patient six months after the hip fracture. The FAC has been used in other studies [6,11,19] and provides a useful measure of the patient’s walking ability. The scale was dichotomized with a cut-off of FAC < 3 and ≥3 to differentiate between patients who required physical assistance from another person and those who did not. The FAC was assessed during outpatient orthopedic follow-up visits at 1, 3, 6, and 12 months, and recovery-related information was collected at each visit. The analysis focused on the 6-month outcomes, as some patients were still recovering at the earlier assessments. No patient showed any additional improvement in recovery between the 6- and 12-month assessments.

The trauma surgeon indicated full or non-full weight-bearing status before the first physiotherapy session. Partial weight-bearing was considered non-full weight-bearing. If full weight-bearing was not allowed, it was typically prohibited for 2–4 weeks after surgery, although this varied.

Short- and long-term survival were recorded. The date of death was obtained from the patient’s medical records and the Ministry of Health’s National Death Index website (https://www.mscbs.gob.es/estadEstudios/estadisticas/estadisticas/estMinisterio/IND_TipoDifusion.htm, accessed on 1 March 2024), with authorization.

Pre-existing depression was analyzed separately because it is a common comorbidity that is not included in the Charlson Comorbidity Index. Furthermore, previous studies have suggested that depression may be independently associated with higher odds of poor functional outcomes [20].

### 2.4. Statistical Analysis

For categorical variables, absolute numbers, relative frequencies, and proportions were calculated and compared using Fisher’s exact test. Continuous variables were presented as means and standard deviations (SD), and compared using Student’s *t*-test. Spearman’s correlation was used to measure the correlation between the FAC scores before and after hip fracture. To determine the independent variables, we conducted a multivariate logistic regression analysis with forward stepwise selection, including all variables that were statistically significant in the univariate analysis. The odds ratio and 95% confidence interval were then calculated. The area under the ROC curve (AUC) was calculated for our final model. The Hosmer–Lemeshow test was used to determine goodness-of-fit. A Kaplan–Meier curve was calculated to estimate post-discharge survival. Statistical analysis was performed using IBM SPSS Statistics for Windows. Version 29.0 (IBM Corp., Armonk, NY, USA). Statistical significance was set at *p* ≤ 0.05 in all cases.

## 3. Results

Between December 2009 and September 2015, one hundred and seventy-five patients older than 95 years were discharged after a hip fracture. Fifteen (8.6%) patients were excluded because they died within one month of discharge, and 8 (4.6%) patients because their walking ability had not been recorded. The remaining 152 patients were included in the analysis. One patient (0.6%) could not be located after the second year. One patient was still alive ten years later at the time of the study. All fractures were caused by low-energy trauma.

Table 1 shows the differences in maximum ambulation ability prior to the fracture and six months after discharge, as measured by the FAC. The mean pre-fracture FAC was 3.12 (SD 1.36) compared to 1.67 (SD 1.64) after discharge. Before the fracture, 78.3% of patients were classified as independent in ambulation (FAC ≥ 3), a figure that decreased to 36.8% six months after discharge. Consequently, of those previously able to walk independently, 46.9% recovered the ability to walk unaided. A positive correlation was found between pre- and post-hip-fracture FAC scores, with rho = 0.492 (*p* < 0.001).

Table 2 and Table 3 compare the baseline characteristics and in-hospital results for exceptional longevity patients classified as FAC < 3 and FAC ≥ 3.

**Figure 1:**
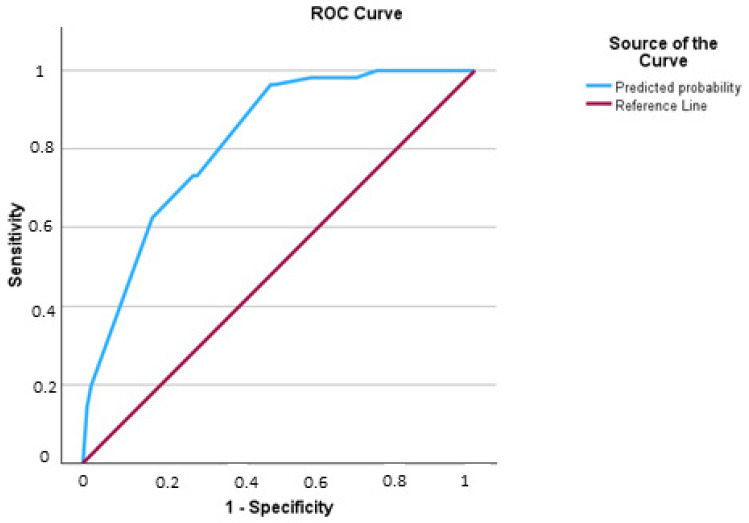
Area under the ROC curve for walking recovery. AUC 0.819 (0.754–0.885), *p* < 0.001.

The results of the multivariate logistic regression analysis revealed that the predictors of walking recovery were a better pre-fracture FAC score (*p* < 0.001, OR 3.658. 95% CI 2.141–6.25), the absence of delirium during hospitalization (*p* = 0.010, OR 3.45. 95% CI 1.342–8.873) and full weight-bearing capacity (*p* = 0.026, OR 12.705. 95% CI 1.362–118.475). The area under the ROC curve for walking recovery was 0.819 (*p* < 0.001, 95% CI 0.754–0.885) Figure 1.

The Hosmer–Lemeshow test showed that the model was well calibrated (*p* = 0.758). Replicating the model with the addition of age and sex yielded very similar results and identified the same predictors.

The distribution of surgical procedures was as follows: hemiarthroplasty in 54 patients (35.5%), cannulated screw fixation in 6 (4.0%), sliding hip screw fixation in 59 (38.8%), and intramedullary nailing in 33 (21.7%). Among the 13 patients who were unable to bear full weight, 7 (53.8%) had an intracapsular fracture and 6 (46.2%) an extracapsular fracture. Four patients (30.8%) underwent unipolar hemiarthroplasty, 2 (15.4%) were treated with cannulated screws, 3 (23.1%) with sliding hip screws, and 4 (30.8%) with intramedullary nails. Orthopedic complications were infrequent. During hospitalization, one patient (0.7%) experienced a hemiarthroplasty dislocation and one (0.7%) sustained cephalic screw failure. During the first 6 months of follow-up, one additional patient (0.7%) experienced a hemiarthroplasty dislocation, one (0.7%) sustained a peri-implant fracture, and two (1.3%) developed surgical site infection. Given the very low incidence of complications, these events were not included in the statistical analysis.

**Figure 2:**
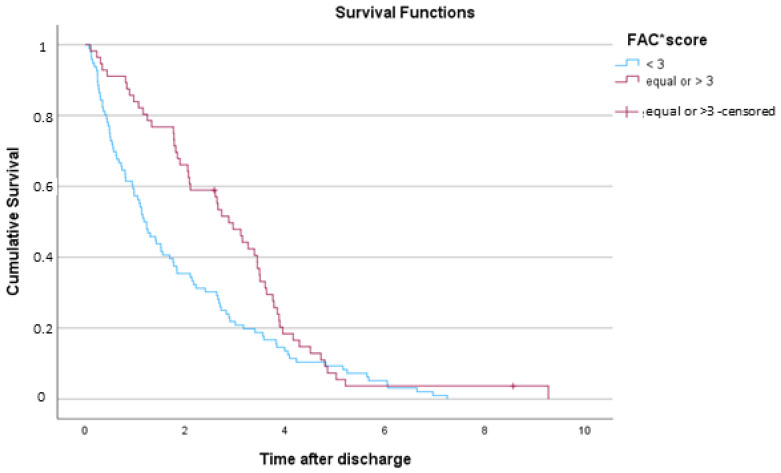
Long-term cumulative survival according to level of walking ability achieved six months after discharge. * Functional ambulation classification.

Survival rates were 99.3% at 1 month, 78.7% at 6 months, 64.5% at 1 year and 7.9% at 5 years. Mean survival after discharge was 2.24 years (SD 1.185). Figure 2 compares the cumulative survival rates of patients who required assistance with ambulation six months after discharge with those who did not. This difference was maintained for approximately four years, after which it leveled off.

## 4. Discussion

This study shows that nearly half the patients aged over 95 years who were walking independently before the fracture were able to walk unassisted 6 months after the hip fracture. The main predictors of walking recovery were better previous walking ability (as measured by the FAC) and full weight-bearing capacity after surgery. Delirium during hospitalization, on the other hand, was associated with poor functional recovery. It is also worth mentioning that despite the very advanced age of patients in our study (mean age 97 years) and the fact that almost one third had dementia, the vast majority (78.3%) had an autonomous gait before the fracture.

The few studies conducted on patients with exceptional longevity show very uneven results regarding walking recovery. In a 2004 study, Oliver et al. reported that 22% of patients regained their pre-fracture walking ability [9]. The worst results were found by Sarasa-Roca et al., with only 2 of 14 patients (12%) who were previously ambulatory recovering the ability to walk [10]. Shabat et al. reported that 4 of 11 patients (36%) regained the ability to walk and achieve ambulation [21]. All three studies were conducted on small numbers of patients. In addition, Holt et al. found, in their prospective study of 50 patients aged 95 years and over who underwent surgery for hip fracture, that 44% regained their walking ability at one year; 96% of these patients were able to walk without assistance before the hip fracture and 40% after hip fracture [11]. A more recent study including 253 centenarians based on data from the Spanish National Hip Fracture Registry showed similar results to ours for one-month walking recovery, with almost 50% of patients regaining independent ambulation [12]. The best results were reported by Barret-Lee et al. in a study of 60 centenarians, in which 63% of patients returned to their pre-fracture level of independence [13].

The risk factors for non-functional recovery have not been described in patients with exceptional longevity. However, Ouellet et al. reported that age, dementia, delirium, number of medications and opiate use were predictors of poor functional outcome at three and six months in patients older than 65 years who had previously been autonomous [22]. In a retrospective study of 228 patients with a follow-up rate of 54%, Takahashi et al. found that FAC score before the fracture and at discharge correlated positively with FAC at 6 months. Age and surgical delay worsened the prognosis but only in patients with hip neck fractures [6]. Pioli et al., in a study of 774 patients divided into three groups according to pre-fracture ambulation capacity (mobile outdoors, mobile indoors, and mobile with assistance) found that different factors predicted walking recovery, depending on the time of assessment (at 3, 6 or 12 months). The only significant predictor in all three groups was pre-fracture functional status [23].

It would seem obvious that patients with a better functional status and better walking ability before the fracture would be the most likely to regain walking ability. Some authors have suggested that physical resilience in one domain can help offset an unfavorable status in others, and that circumstances that are not always considered, such as living situation (e.g., at home or in an institution) and socio-economic status, may be more important in some patients than disease burden [24].

Full weight-bearing capacity is an understudied aspect of functional recovery after hip fracture. The decision to allow full weight-bearing is made by the traumatologist based on their experience and the stability of the fracture after the surgery. Non-full weight-bearing typically lasts only a few days or weeks, but generally starts early in rehabilitation. In a similar study to ours with a longer follow-up period, Ariza-Vega et al. found that full weight-bearing was an independent predictor of one-year functional outcome, together with the patient’s pre-fracture functional status, cognitive status, health status, age and fracture type [25]. In a prospective study of 41 patients with pertrochanteric hip fractures, divided in two groups with similar baseline characteristics, Pfeufer et al. found that weight-bearing restrictions in older adults contributed to loss of mobility and slower gait speed when measured on the fifth post-operative day [26]. However, in a retrospective study in Switzerland of 219 patients over 70 years of age, the authors found no relationship between full weight-bearing and ability to walk during hospitalization [27].

Despite the increasing use of preventive measures, delirium remains very common among patients admitted for hip fractures. It occurs mainly in patients with previous cognitive impairment, multimorbidity, malnutrition and frailty [28,29]. Advanced age is also a well-known risk factor for delirium, although, again, there are very few studies of extremely older patients admitted with hip fracture. Two studies with small numbers of patients found that approximately 40% of centenarian patients developed delirium during their hospital stays [30], which is consistent with our results, and higher than the 11% reported for younger patients aged 75 to 83 years [31]. Delirium has often been an independent risk factor associated with poor functional recovery after hip fracture in patients in the most frequent age range. Marcantonio et al. reported a decline in ambulation and activities of daily living (ADLs) in hospitalized patients with delirium one month after discharge, and after adjusting for other risk factors such as age, comorbidities and cognitive and functional impairment [32]. A study with a 2-year follow-up comparing patients with and without delirium during hospitalization showed that a greater proportion of the non-delirious group had regained mobility at one year (51% vs. 34%) and this pattern was maintained for 24 months after discharge [33]. Our results in patients with exceptional longevity are consistent with these findings, and with those from other studies describing different forms of cognitive impairment as a risk factor for failure to achieve autonomous walking after hip fracture [34,35]. To our knowledge, there are no previous studies linking delirium and functional recovery in patients with exceptional longevity. In our study, both delirium and dementia were associated with worse outcomes on univariate analysis, but delirium was a better predictor. Delirium probably allowed us to detect those patients with underdiagnosed cognitive impairment, which is common in this very old population.

Thus, our final model included full weight-bearing ability after surgery, pre-fracture walking ability and absence of delirium as predictors of walking recovery, demonstrating good diagnostic performance [36]. If validated in other samples, this could be a useful prognostic model in clinical practice. In a 2021 study of more than 25,000 patients, the authors created a predictive model of gait recovery that included some of the same variables as ours: weight-bearing capacity, cognitive impairment and pre-fracture ambulation. However, their model also included age, ASA score, surgical delay, discharge destination, fracture type and pressure ulcers [8]. In our smaller sample of patients with exceptional longevity, neither age, surgical delay nor fracture type were associated with worse ambulation outcome. Discharge to a center was not significant in the multivariate analysis, but was associated with autonomous walking recovery in the univariate analysis. This may be because only patients who have the best chance of regaining the ability to walk are discharged to a rehabilitation center. Additionally, almost half of the patients who needed assistance to walk resided in nursing homes prior to the fracture.

Patients able to ambulate independently (FAC ≥ 3) showed better short- and long-term survival. In our study, we dichotomized the FAC, as other authors have done, to differentiate between patients requiring human physical support when walking (FAC 0–2) and those able to walk independently without human physical support (FAC 3–5) [37]. A FAC of less than 3 is the cutoff point associated with increased 30-day mortality [19]. Other studies of younger patients have examined this relationship and also found better survival in patients who regained function. A study with 10-year follow-up conducted in Greece with patients with a mean age of 79 years and previously independent, showed that early post-hip fracture walking ability predicted better short-term and long-term survival [15]. In other pathologies requiring hospitalization in older adults, good functional status has been shown to be a protective factor against mortality [38].

It is also worth mentioning that, although these patients were of extremely advanced age, their Charlson index was relatively low. A study conducted in the United Kingdom found no significant differences between centenarians and patients aged 75 to 85 years old [39]. Pelavski et al. suggest that this may be because only the healthiest patients reach 100 years of age [31].

Age was not a statistically significant predictor of the outcome. This is a common finding in very elderly cohorts (≥85–90 years). Although increasing age is often associated with poorer outcomes, when the study population is composed exclusively of very old patients, the independent effect of chronological age may be obscured by other determinants of recovery, such as frailty, pre-fracture functional status, cognitive impairment, and comorbidity burden [40].

Although surgical technique plays an important role in fracture reduction and the likelihood of regaining walking ability [41], it did not appear to be associated with functional recovery in our cohort.

The main limitation of our study is that it was conducted in a single hospital. However, patients were included consecutively, and the number of patients included was higher than in most studies involving this age group. Some patients were excluded because their walking ability had not been recorded, or because they died within one month after discharge and therefore before they could be assessed. These exclusions may have introduced bias into the study. A high percentage of the patients were female (88.2%). This is common among patients with exceptional longevity and reflects reality rather than representing a bias. The study’s main strengths are its description of predictors of walking recovery, and the relationship between walking recovery and short- and long-term survival in patients with exceptional longevity. To our knowledge, this is the first time that these factors have been described in this age group.

## 5. Conclusions

Almost half of patients with exceptional longevity achieved independent ambulation after a hip fracture. Predictors of walking recovery were previous unassisted walking ability and full weight-bearing capacity after surgery. Delirium, on the other hand, was associated with a poor outcome. Recovery of walking ability was a major determinant of both short- and long-term survival in patients with exceptional longevity after hip fracture. These findings highlight the importance of functional outcomes when assessing prognosis in the oldest-old.

## Figures and Tables

**Table 1:** FAC * scores for pre-fracture ambulation and maximum walking ability six months after discharge.

Patients = 152	Before Hip Fracture	6 Months Post-Discharge
FAC * 0 *n* (%)	13 (8.6)	60 (39.5)
FAC * 1 *n* (%)	10 (6.6)	19 (12.5)
FAC * 2 *n* (%)	10 (6.6)	17 (11.2)
FAC * 3 *n* (%)	47 (30.9)	28 (18.4)
FAC * 4 *n* (%)	57 (37.5)	23 (15.1)
FAC * 5 *n* (%)	15 (9.9)	5 (3.3)
Dichotomized FAC		
FAC * < 3 *n* (%)	33 (21.7)	96 (63.2)
FAC * ≥ 3 *n* (%)	119 (78.3)	56 (36.8)

* Functional ambulation classification.

**Table 2:** Comparison of patients older than 95 years requiring physical assistance from another person and those who did not six months after discharge: Baseline characteristics.

Patients = 152	Total *n* = 152	FAC * Score < 3 *n* = 96	FAC * Score ≥ 3 *n* = 56	*p* Value
Age, in years (SD)	97.23 (2.38)	97.50 (2.55)	96.77 (1.98)	0.051
Female sex *n* (%)	134 (88.2)	87 (90.6)	47 (83.9)	0.165
Charlson index (SD)	1.11 (1.1)	1.26 (1.1)	0.86 (1.06)	**0.029**
Pre-fracture FAC * score ≥ 3 *n* (%)	119 (78.3)	63 (65.6)	56 (100)	**<0.001**
Dementia *n* (%)	47 (30.9)	39 (40.6)	8 (14.3)	**<0.001**
Depression *n* (%)	25 (16.4)	20 (20.8)	5 (8.9)	**0.043**
Place of residence: *n* at home (%)	89 (58.6)	47 (49)	42 (75)	**0.001**
Number of drugs (SD)	5 (2.92)	5.58 (2.91)	4 (2.68)	**<0.001**
Intracapsular fracture *n* (%)	68 (44.7)	42 (43.8)	26 (46.4)	0.439
Hemoglobin at admission in g/L (SD)	119.59 (14.96)	118.22 (15.27)	122.02 (14.22)	0.133
Albumin at admission in g/L (SD)	28.24 (3.23)	28.39 (3.1)	27.99 (3.46)	0.485

Note: SD = standard deviation. * Functional ambulation classification.

**Table 3:** Comparison of patients older than 95 years requiring physical assistance from another person and those who did not six months after discharge: In-hospital test results.

Patients = 152	Total *n* = 152	FAC * Score < 3 *n* = 96	FAC * Score ≥ 3 *n* = 56	*p* Value
Length of hospital stay, in days (SD)	12.69 (8.42)	13.56 (10.05)	11.20 (4.04)	**0.043**
Delay to surgery, in days (SD)	3.60 (2.44)	3.81 (2.63)	3.23 (2.05)	0.133
Prosthesis *n* (%)	54 (35.5)	32 (33.3)	22 (39.3)	0.285
Uncemented unipolar *n*/54 (%)	41 (75.9)	26 (81.3)	15 (68.2)	0.217
Cemented bipolar *n*/54 (%)	13 (24.1)	6 (18.8)	7 (31.8)	
Spinal anesthesia *n* (%)	148 (97.4)	92 (95.8)	56 (100)	0.155
Transfusion *n* (%)	92 (60.5)	61 (63.5)	31 (55.4)	0.205
Delirium *n* (%)	42 (27.6)	32 (33.3)	10 (17.9)	**0.029**
Patients with medical complications *n* (%)	71 (46.7)	51 (53.1)	20 (35.7)	**0.028**
Patients with traumatological complications *n* (%)	2 (1.3)	1 (1)	1 (1.8)	0.603
Physiotherapy: full weight-bearing *n* (%)	139 (91.4)	84 (87.5)	55 (98.2)	**0.018**
Skin or respiratory isolation *n* (%)	2 (1.3)	2 (2.1)	0 (0)	0.397
Discharge to a center *n* (%)	64 (42.1)	35 (36.5)	29 (51.8)	**0.047**

Note: SD = standard deviation. * Functional ambulation classification.

## Data Availability

The datasets used in this study are available from the corresponding author upon reasonable request.

## References

[1] Teixeira L., Araújo L., Jopp D., Ribeiro O. (2017). Centenarians in Europe. Maturitas.

[2] Rodríguez-Molinero A., Yuste A., Banegas J.R. (2010). High incidence of hip fracture in Spanish centenarians. J. Am. Geriatr. Soc..

[3] Rosell P.A., Parker M.J. (2003). Functional outcome after hip fracture. A 1-year prospective outcome study of 275 patients. Injury.

[4] Kammerlander C., Gosch M., Kammerlander-Knauer U., Luger T.J., Blauth M., Roth T. (2011). Long-term functional outcome in geriatric hip fracture patients. Arch. Orthop. Trauma Surg..

[5] González Marcos E., González García E., González-Santos J., González-Bernal J.J., Del Pilar Martín-Rodríguez A., Santamaría-Peláez M. (2022). Determinants of Lack of Recovery from Dependency and Walking Ability Six Months After Hip Fracture in a Population of People Aged 65 Years and Over. J. Clin. Med..

[6] Takahashi A., Naruse H., Kitade I., Shimada S., Tsubokawa M., Kokubo Y., Matsumine A. (2020). Functional outcomes after the treatment of hip fracture. PLoS ONE.

[7] Hannan E.L., Magaziner J., Wang J.J., Eastwood E.A., Silberzweig S.B., Gilbert M., Morrison R.S., McLaughlin M.A., Orosz G.M., Siu A.L. (2001). Mortality and locomotion 6 months after hospitalization for hip fracture: Risk factors and risk-adjusted hospital outcomes. JAMA.

[8] González de Villaumbrosia C., Sáez López P., Martín de Diego I., Lancho Martín C., Cuesta Santa Teresa M., Alarcón T., Ojeda Thies C., Queipo Matas R., González-Montalvo J.I., on behalf of the Participants in the Spanish National Hip Fracture Registry (2021). Predictive Model of Gait Recovery at One Month After Hip Fracture from a National Cohort of 25,607 Patients: The Hip Fracture Prognosis (HF-Prognosis) Tool. Int. J. Environ. Res. Public Health.

[9] Oliver C.W., Burke C. (2004). Hip fractures in centenarians. Injury.

[10] Sarasa-Roca M., Torres-Campos A., Redondo-Trasobares B., Angulo-Castaño M.C., Gómez-Vallejo J., Albareda-Albareda J. (2022). Hip fracture in centenarians, ¿what can we expect?. Rev. Esp. Cir. Ortop. Traumatol..

[11] Holt G., Macdonald D., Fraser M., Reece A.T. (2006). Outcome after surgery for fracture of the hip in patients aged over 95 years. J. Bone Jt. Surg..

[12] Bermejo Boixareu C., Ojeda-Thies C., Guijarro Valtueña A., Cedeño Veloz B.A., Gonzalo Lázaro M., Navarro Castellanos L., Queipo Matas R., Gómez Campelo P., Royuela Vicente A., González-Montalvo J.I. (2023). Clinical and Demographic Characteristics of Centenarians versus Other Age Groups Over 75 Years with Hip Fractures. Clin. Interv. Aging.

[13] Barrett-Lee J., Barbur S., Johns J., Pearce J., Elliot R.R. (2021). Hip fractures in centenarians: A multicentre review of outcomes. Ann. R. Coll. Surg. Engl..

[14] Lari A., Haidar A., AlRumaidhi Y., Awad M., AlMutairi O. (2022). Predictors of mortality and length of stay after hip fractures—A multicenter retrospective analysis. J. Clin. Orthop. Trauma..

[15] Iosifidis M., Iliopoulos E., Panagiotou A., Apostolidis K., Traios S., Giantsis G. (2016). Walking ability before and after a hip fracture in elderly predict greater long-term survivorship. J. Orthop. Sci..

[16] Barceló M., Torres O., Ruiz D., Casademont J. (2018). Hip Fractures in People Older Than 95 Years: Are Patients Without Age-Associated Illnesses Different?. J. Gerontol. A Biol. Sci. Med. Sci..

[17] Charlson M.E., Pompei P., Ales K.L., MacKenzie C.R. (1987). A new method of classifying prognostic comorbidity in longitudinal studies: Development and validation. J. Chronic Dis..

[18] Holden M.K., Gill K.M., Magliozzi M.R., Nathan J., Piehl-Baker L. (1984). Clinical gait assessment in the neurologically impaired: Reliability and meaningfulness. Phys. Ther..

[19] López-Torres I.I., Sanz-Ruiz P., Montero-Fernández N., Chana F., Serra-Rexach J.A., Benjumea-Carrasco A., Vaquero-Martín J. (2020). Surgical treatment of hip fracture in centenarians: Complications and independent risk factors of death. Injury.

[20] Remelli F., Ferrara M.C., Triolo F., Belvederi Murri M., Caruso G., Belleli G., Volpato S., Trevisan C. (2024). Depression and Functional Recovery After Hip Fracture in Community-Dwelling Older Adults. J. Frailty Aging.

[21] Shabat S., Mann G., Gepstein R., Fredman B., Folman Y., Nyska M. (2004). Operative treatment for hip fractures in patients 100 years of age and older: Is it justified?. J. Orthop. Trauma.

[22] Ouellet J.A., Ouellet G.M., Romegialli A.M., Hirsch M., Berardi L., Ramsey C.M., Cooney L.M., Walke L.M. (2019). Functional Outcomes After Hip Fracture in Independent Community-Dwelling Patients. J. Am. Geriatr. Soc..

[23] Pioli G., Lauretani F., Pellicciotti F., Pignedoli P., Bendini C., Davoli M.L., Martini E., Zagatti A., Giordano A., Nardelli A. (2016). Modifiable and non-modifiable risk factors affecting walking recovery after hip fracture. Osteoporos. Int..

[24] Ek S., Wennberg A.M., Ding M., Meyer A.C., Hedström M., Modig K. (2024). Characterizing the Individuals Who Regain or Maintain Walking Ability After a Hip Fracture: Insights Into Physical Resilience. J. Am. Med. Dir. Assoc..

[25] Ariza-Vega P., Jiménez-Moleón J.J., Kristensen M.T. (2014). Non-weight-bearing status compromises the functional level up to 1 yr after hip fracture surgery. Am. J. Phys. Med. Rehabil..

[26] Pfeufer D., Zeller A., Mehaffey S., Böcker W., Kammerlander C., Neuerburg C. (2019). Weight-bearing restrictions reduce postoperative mobility in elderly hip fracture patients. Arch. Orthop. Trauma Surg..

[27] Baer M., Neuhaus V., Pape H.C., Ciritsis B. (2019). Influence of mobilization and weight bearing on in-hospital outcome in geriatric patients with hip fractures. SICOT J..

[28] Chen Y., Liang S., Wu H., Deng S., Wang F., Lunzhu C., Li J. (2022). Postoperative delirium in geriatric patients with hip fractures. Front. Aging Neurosci..

[29] Koskderelioglu A., Onder O., Gucuyener M., Altay T., Kayali C., Gedizlioglu M. (2017). Screening for postoperative delirium in patients with acute hip fracture: Assessment of predictive factors. Geriatr. Gerontol. Int..

[30] Konttinen N., Rosenberg P.H. (2006). Outcome after anaesthesia and emergency surgery in patients over 100 years old. Acta Anaesthesiol. Scand..

[31] Pelavski Atlas A.D., Colomina M.J., De Miguel M., Roigé J. (2009). Centenarian versus patients within the most frequent age range for hip fractures: Transfusion practice. Ach Orthop. Trauma Surg..

[32] Marcantonio E.R., Flacker J.M., Michaels M., Resnick N.M. (2000). Delirium is independently associated with poor functional recovery after hip fracture. J. Am. Geriatr. Soc..

[33] Dolan M.M., Hawkes W.G., Zimmerman S.I., Morrison R.S., Gruber-Baldini A.L., Hebel J.R., Magaziner J. (2000). Delirium on hospital admission in aged hip fracture patients: Prediction of mortality and 2-year functional outcomes. J. Gerontol. A Biol. Sci. Med. Sci..

[34] Loh Y.L., Wicks J., Alexander T. (2023). The impact of dementia on rehabilitation outcomes following hip fracture. Aging Med..

[35] Ariza-Vega P., Lozano-Lozano M., Olmedo-Requena R., Martín-Martín L., Jiménez-Moleón J.J. (2017). Influence of Cognitive Impairment on Mobility Recovery of Patients with Hip Fracture. Am. J. Phys. Med. Rehabil..

[36] Çorbacıoğlu Ş.K., Aksel G. (2023). Receiver operating characteristic curve analysis in diagnostic accuracy studies: A guide to interpreting the area under the curve value. Turk. J. Emerg. Med..

[37] Hammer A., Ljungberg K., Bohman T., Andersson Å.G. (2022). Description and comparison of postoperative functioning of patients with hip fracture 2018 and 2008 at the Örebro University Hospital—A comparative cross-sectional study. BMC Geriatr..

[38] Torres O.H., Muñoz J., Ruiz D., Ris J., Gich I., Coma E., Gurguí M., Vázquez G. (2004). Outcome predictors of pneumonia in elderly patients: Importance of functional assessment. J. Am. Geriatr. Soc..

[39] Verma R., Rigby A.S., Shaw C.J., Mohsen A. (2009). Acute care of hip fractures in centenarians—Do we need more resources?. Injury.

[40] Arinzon Z., Fidelman Z., Zuta A., Peisakh A., Berner Y.N. (2005). Functional recovery after hip fracture in old-old elderly patients. Arch. Gerontol. Geriatr..

[41] Cibula Z., Grendar M., Sammoudi D., Cipkala M., Melisik M., Hrubina M. (2025). Cement Augmentation of the Blade in Proximal Femoral Nailing for Trochanteric Fractures in Elderly Patients: A Retrospective Comparison of Mechanical Stability and Complications. J. Clin. Med..

